# PRECARITY AND LATER LIFE: UNDERSTANDING NEW INEQUALITIES AND RISKS IN LATER LIFE

**DOI:** 10.1093/geroni/igz038.2181

**Published:** 2019-11-08

**Authors:** Dale Dannefer, Christopher Phillipson, Dale Dannefer

**Affiliations:** 1 Department of Sociology, Case Western Reserve University, Cleveland, Ohio, United States; 2 The University of Manchester, Manchester, United Kingdom

## Abstract

This symposium addresses debates around the theme of precarity and its implications for understanding social and economic changes affecting the lives of older people. To date, the concept of precarity has been applied to several subpopulations by various academic disciplines but has yet to be systematically applied to later life. The symposium will give particular attention to the extent to which the lens provided by precarity can illuminate different types of inequalities experienced through the life course and reflected in public policies directed at older people. Chris Phillipson reviews theoretical perspectives relating to precarity, examining their potential contribution for the development of critical gerontology. His paper also considers the extent to which the concept of ‘precarious ageing’ offers a competing or complementary view to theories of ‘active’ and ‘successful ageing’. Larry Polivka examines the growing precarity of life for older Americans emanating from austerity budgets and privatization of public services. The paper suggests that policies such as health care and long term care are in jeopardy, creating a glide path toward the extension of precarious employment into a precarious retirement for millions of older people. Wenxuan Huang examines how the focus on agency and other individual-level foci obscure understanding of social dynamics. Finally, Amanda Grenier draws on a scoping review of precarity to outline conceptual distinctions between frailty, vulnerability, and precarity. She presents reflections on what these concepts offer in terms of understandings of late life the study of disadvantage across the life course.

